# Sickness in High Altitude

**Published:** 1901-03

**Authors:** C. E. Boynton

**Affiliations:** Sandy, Utah


### CORRESPONDENCE.

### SICKNESS IN HIGH ALTITUDE.

Whenever a disease overworks the heart it may better be treated at sea-level than in the upper air of a mountain region. All forms of heart disease are more rebellious to treatment in high altitudes. From the State of Utah it is about eight hundred miles to sea-level in any direction, besides the journey is quite expensive.

At the present time I am treating a young lady of twenty-five years who has for eleven years suffered with valvular disease. Up here she is an incurable, but at sea-level I believe she could enjoy life for many years. I say this because I have practised several years in both situations and at various altitudes. But a transfer of home to sea-level is out of the question in this case because of deficient finances. I could enumerate dozens of cases in proof of the well known fact that incurable valvular disease of high altitude rapidly improves at sea-level; but in sending a patient away we must be careful that we do not send them to a malarial district. There is very little malaria in Utah ; but Utah people are highly susceptible to malaria in other situations.

C. E. Boynton, M.D.

*Sandy, Utah.*

